# Assessment of Deep Learning Models for Cutaneous Leishmania Parasite Diagnosis Using Microscopic Images

**DOI:** 10.3390/diagnostics14010012

**Published:** 2023-12-20

**Authors:** Ali Mansour Abdelmula, Omid Mirzaei, Emrah Güler, Kaya Süer

**Affiliations:** 1 Department of Microbiology and Clinical Microbiology, Faculty of Medicine, Near East University, North Cyprus, Mersin 10, Lefkoşa 99010, Turkey; 2 Department of Biomedical Engineering, Faculty of Engineering, Near East University, North Cyprus, Mersin 10, Lefkoşa 99010, Turkey; omid.mirzaei@neu.edu.tr; 3 Research Center for Science, Technology and Engineering (BILTEM), Near East University, TRNC, Mersin 10, Lefkoşa 99138, Turkey; 4 Department of Molecular Biology and Genetics, Faculty of Arts and Sciences, European University of Lefke, Lefke 99010, Turkey; eguler@eul.edu.tr; 5 Department of Clinical Microbiology and Infectious Diseases, Faculty of Medicine, Near East University, North Cyprus, Mersin 10, Lefkoşa 99010, Turkey; kaya.suer@neu.edu.tr

**Keywords:** cutaneous leishmaniasis, amastigotes stage, CNNs, deep learning

## Abstract

Cutaneous leishmaniasis (CL) is a common illness that causes skin lesions, principally ulcerations, on exposed regions of the body. Although neglected tropical diseases (NTDs) are typically found in tropical areas, they have recently become more common along Africa’s northern coast, particularly in Libya. The devastation of healthcare infrastructure during the 2011 war and the following conflicts, as well as governmental apathy, may be causal factors associated with this catastrophic event. The main objective of this study is to evaluate alternative diagnostic strategies for recognizing amastigotes of cutaneous leishmaniasis parasites at various stages using Convolutional Neural Networks (CNNs). The research is additionally aimed at testing different classification models employing a dataset of ultra-thin skin smear images of Leishmania parasite-infected people with cutaneous leishmaniasis. The pre-trained deep learning models including EfficientNetB0, DenseNet201, ResNet101, MobileNetv2, and Xception are used for the cutaneous leishmania parasite diagnosis task. To assess the models’ effectiveness, we employed a five-fold cross-validation approach to guarantee the consistency of the models’ outputs when applied to different portions of the full dataset. Following a thorough assessment and contrast of the various models, DenseNet-201 proved to be the most suitable choice. It attained a mean accuracy of 0.9914 along with outstanding results for sensitivity, specificity, positive predictive value, negative predictive value, F1-score, Matthew’s correlation coefficient, and Cohen’s Kappa coefficient. The DenseNet-201 model surpassed the other models based on a comprehensive evaluation of these key classification performance metrics.

## 1. Introduction

The Trypanosomatidae family consists of single-celled, obligate parasites that infect invertebrates, vertebrates, and plants. These eukaryotic flagellates possess a distinctive single mitochondrion DNA known as kinetoplast, which is a defining characteristic of the Kinetoplastea Class. The positioning of the kinetoplast in relation to the nucleus and the flagella’s point of emergence allows for the identification of specific life cycle forms, which are unique to certain genera within the family [1,2]. Cutaneous Leishmaniasis is a disease that is frequently found in tropical and subtropical areas and is transmitted by this particular protozoan species. The bite of an infected sandfly spreads this animal protozoan [3]. Despite its prevalence throughout the world, most cases are found in South America, the Mediterranean basins, and some parts of Asia and Africa [4]. The Caribbean, the Mediterranean basins, the Arabian Peninsula, and Central Asia account for about 95% of all CL cases [5]. The World Health Organization (WHO) estimates that between 700,000 and 1,000,000 new cases are reported each year. Malnutrition, population dislocation, inadequate housing, unhygienic circumstances, a compromised immune system, and a shortage of resources are all associated factors [6]. The inoculation site is usually an exposed part of the body, such as the scalp and extremities, where both of these groups normally form tiny pustules [7]. Infection with the Leishmania parasite can result in cutaneous leishmaniasis (CL), an infection of the skin that can occur in people who have been bitten by infected sand flies. There have also been reports of transmission between humans via infected acupuncture needles, transfusions of blood, or prenatal transmission [7,8]. The types and severity of skin infections associated with this condition vary, and they can typically appear many weeks or even months after infection [9]. Papules gradually enlarge into nodules that become ulcerated [10]. The last ulcer, a hallmark of CL, self-heals in three to eighteen months, depending on the species. According to estimates, up to 10% of CL cases develop, become chronic, and display increasingly severe clinical symptoms [7,11,12]. Despite the fact that CL rarely results in death, it can have a detrimental influence on quality of life and cause significant morbidity [13]. A recent genetic investigation in Libya revealed that parasites such as *Leishmania major* and *Leishmania tropica* are the primary cause of CL in the nation. Additionally, the illnesses identified in Libya’s various regions substantially reflect those observed in CL-affected Mediterranean locales.

Artificial intelligence (AI) principles have enormous potential for a wide range of applications, including risk modeling and classification, self-detection, diagnostics, including the classification of small molecules into illness subgroups, and the prediction of treatment response and prognosis. AI is increasingly being used in medical and biological research as well as therapeutic treatment [14,15]. Several preclinical and clinical studies on healthcare have been conducted recently using supervised machine learning (SML) and various AI technologies. In terms of e-health care, particularly the accurate identification and classification of diseases, SML has changed almost every industry internationally. For many aspects of healthcare, multiple university and industry labs are creating AI technologies [16]. There are numerous applications based on medical imaging investigations, including the identification and diagnosis of squamous cell carcinoma [17] and lung diseases [18]. Medical image analysis has recently benefited greatly from deep learning applications [19].

Convolutional neural networks (CNNs) have produced exceptional classification and segmentation results for images. Clinical prediction frameworks have been developed, and significant linkages have been explained thanks to the application of data analysis, machine learning, and deep learning algorithms in modern healthcare [20,21]. A CNN is a deep training algorithm that primarily focuses on object and image classification algorithms [22]. The first layer of a convolutional neural network is composed of the convolutional layer and the pooling layer together, while the final layer is the fully connected (FC) layer [23]. The density between these layers can be scaled up to capture more fine detail, but doing so will require more computer power depending on the complexity of the images [24,25]. The foundational component of CNN is where the majority of computations take place. After the first conversion layer, there could be a second conversion layer. During the convolution process, a kernel or filter within this particular layer shifts throughout the image’s receptive fields to assess whether a characteristic is present [26,27]. The input parameter count is decreased by the pooling layer, but some data are also lost as a result. Positively, this layer streamlines operations and increases the CNN’s effectiveness [28]. Complete connection refers to the connection of all inputs or nodes from one layer to each activated unit or cluster from the following layer [29].

Microbiologists continue searching for novel microbiology diagnostic procedures that are more rapid, less expensive, more accurate, and treatment-oriented due to the shortcomings of conventional diagnostic performances. Due to variations in species and sizes, the physical detection of leishmaniasis can be laborious, slow, and inaccurate. Expertise is required for precise detection, which is more challenging in complex circumstances. This study’s primary objective is to assess alternative diagnostic approaches for the cutaneous leishmaniasis parasites using a convolutional neural network model.

- The implementation of pre-trained models (EfficientNet-B0, DenseNet201, Mobilenet-v2, ResNet101, and Xception) for the classification of microscopic images as positive and negative is one of the study’s key achievements;
- Performance evaluation of the models can be assessed using various metrics, including accuracy, sensitivity, specificity, precision, F1-Score, Matthew’s correlation coefficient (MCC), Negative Predictive Value (NPV), Cohen’s kappa, Area Under Curve (AUC), and Receiver Operating Characteristic (ROC) curve;
- One of the purposes of this investigation is to evaluate and compare the efficiency of five different pre-trained deep-learning models within the realm of classification tasks.

## 2. Related Work

In this study, focusing on the classification of cutaneous leishmaniasis, microscopic images are mostly taken into consideration. In this section, studies in the literature are presented in detail.

### 2.1. Cutaneous Leishmaniasis

The study used microscopic images to analyse diverse cultures of cutaneous leishmania parasites. With diameters of roughly 1500–1300 pixels, the images caught and annotated the promastigote and amastigote stages of *Leishmania infantum*, *Leishmania major*, and *Leishmania braziliensis*. The work was a success in terms of creating parasite cultures, annotating images, and training the U-Net model. During training, a U-Net model was used for pixel-wise classification to solve class imbalances. Performance was evaluated using criteria such as precision, recall, and F1-score. The approach used to deal with class imbalance was critical for analysing pixel percentages per class and revealed information on image region distribution and representation. The algorithm used accurately detected 82.3% of amastigote stage frequencies [30]. Using a dataset of 300 images gathered from 50 laboratory slides, this study attempted to develop an artificial intelligence-based technique to perform the automated diagnosis of leishmaniasis. These slides were obtained from patients at the Valfajr Clinic in Shiraz, Iran, and included 150 images from positive and 150 from negative leishmania slides. The Viola–Jones technique was used in the algorithm, which included three important steps: feature extraction, integral picture creation for quicker processing, and classification using Haar-like features. The classifier was trained using the AdaBoost algorithm after discriminative characteristics were chosen. The task of recognising amastigotes outside of macrophages had a recall of 0.520 and a precision of 0.711 [31]. The images have been captured with a smartphone at a microscopic magnification of 50 and exported in PNG format with an average resolution of 1320 px × 1900 px. The collection was 45 microscopic images in total. The resulting images were pre-processed, and pre-training was implemented for segmentation images of promastigotes and amastigotes forms for cutaneous leishmanials using the K-means algorithm, histogram thresholding, and the U-net structure. Individual precision and recall values for amastigotes phases were 61.07% and 87.90%, respectively [32].

### 2.2. Plasmodium Parasites (Malaria)

For the all-important work of identifying malaria by blood smear testing, 27,558 malaria blood smear images from the National Institute of Health (NIH) and expanded wielding rotation, zooming, and flipping, then a two-stage technique was created. Initially, a U-Net method was used to precisely segment red blood cell clusters. Following that, faster R-CNN was used to recognise smaller cell objects inside these linked components. This technique was especially successful because of its versatility, with U-Net-derived cell-cluster masks guiding the detection process, resulting in higher true positive rates and lower false alarms. A unique CNN termed Attentive Dense Circular Net (ADCN) was introduced for the successful classification of malaria-infected red blood cells, inspired by residual and dense networks and including an attention mechanism. The revolutionary inclusion of attention mechanisms in ADCN allows it to focus on key features, resulting in a patient-level accuracy of 97.47% in the classification of infected RBCs [33,34]. Pattanaik and colleagues introduced a novel approach, the Multi-Magnification Deep Residual Network (MM-ResNet), tailored for the classification of malaria-infected blood smears captured using Android smartphones. Their study utilised a publicly available dataset consisting of 1182 field-stained images, encompassing three magnification levels: 200×, 400×, and 1000×. MM-ResNet is built upon convolutional layers, batch normalization, and ReLU activation functions, trained with a single pass through the data, reducing the data requirements. This model effectively mitigates the challenges posed by the low image quality, varying luminance, and noise inherent in smartphone-captured images, thanks to the utilization of residual connections and an abundance of filters. Remarkably, the proposed MM-ResNet achieved an impressive accuracy rate of 98.08% during five-fold cross-validation, demonstrating its efficacy in malaria blood smear classification across varying magnifications and challenging image conditions [35].

### 2.3. Trypanosoma Parasites

Traditional approaches for identifying Chagas disease’s acute phase entail detecting *Trypanosoma cruzi* in peripheral blood slides using microscopy. An innovative approach analyses image tiles from these samples using MobileNet V2 convolutional layers, yielding 1280-dimensional feature vectors that are input into a single-neuron classifier. Initial validation tests on a small 12-slide dataset achieved 96.4% accuracy but dipped to 72.0% on the 13th slide. Incorporating images from six additional slides, including thick blood samples, raised the accuracy to 95.4% on two further slides. Raster scans with overlapping windows efficiently reveal positive *Trypanosoma cruzi* occurrences in both blood smear and thick blood images, highlighting the method’s potential to boost Chagas disease detection [36]. In this study, an innovative approach was developed for the automatic detection of the *Trypanosoma cruzi* parasite in blood smears using machine learning techniques applied to mobile phone images. A total of 33 slides containing thin blood smears from Swiss mice infected with the T. cruzi Y strain during the acute phase were analyzed. Images were standardised to 768 × 1024 pixels^2^, and parasites were segmented, with 100 × 100 pixels^2^ regions around each parasite cropped based on manual annotations. Object features were extracted after converting from RGB to CIEL colour space. To enhance performance and mitigate noise, Principal Component Analysis (PCA) was applied. Supervised learning classifiers, such as Support Vector Machines (SVM), K-nearest neighbors (KNN), and Random Forest (RF), were employed due to their strong generalization with limited data. The dataset was split into training and test sets (4:1 ratio) and classified using the RF algorithm. The proposed method demonstrated significant precision (87.6%), sensitivity (90.5%), and an impressive Area Under the Receiver Operating Characteristic (ROC) Curve of 0.942. This research showcases the potential of automating image analysis through mobile devices, offering a cost-effective and efficient alternative to traditional optical microscope methods [37]. A mobile bot application was created using deep learning to identify *Trypanosoma evansi* infection using 125 images of *T. evansi* obtained in an oil immersion field. In a one-stage learning technique, the YOLOv4-tiny model was used to categorise two particular parasite stages: “*Trypanosoma_evansi*_slender” and “*Trypanosoma_evansi*_stumpy”. The addition of 20-degree rotational angles to the data resulted in remarkable performance metrics: 95% sensitivity, specificity, precision, accuracy, and F1 score with a misclassification rate of less than 5%. CIRA Bot, the simulation platform, produced results equivalent to those of computational trials, with an area under the ROC curve of 0.964 and a precision-recall curve of 0.962. This breakthrough offers considerable potential for using thin-blood film evaluation to diagnose *T. evansi* infection [38].

### 2.4. Cryptococcus neoformans

Web scraping was used in this study to collect data on microscopic images of both *C. neoformans* and non-*C. neoformans* using specified keywords such as “India-ink-stained smear of CSF”, “*C. neoformans*”, and others. These images were classified as positive or negative, yielding a dataset of 63 high-quality microscopic images for each category, which was later expanded to 1000 images. The study is supposed to recognise and classify *C. neoformans* images using a deep learning strategy based on convolutional neural networks (CNN). Various frameworks and libraries were used to carry out model training, validation, testing, and evaluation. The VGG16 model, deemed cutting-edge, performed successfully, with an accuracy of 86.88% and a loss of 0.36203, respectively [39].

Artificial intelligence is not only used for medical issues; fingerprinting is another application of biometrics used for personal identification due to its uniqueness and individualistic characteristics. However, traditional algorithms encounter difficulties when recreating fingerprint images due to poor quality and structured noise. This paper introduces a novel fingerprint system that employs a sparse autoencoder (SAE) algorithm to reconstruct fingerprint images. The SAE, an unsupervised deep learning model, replicates its input at the output. The model is trained and optimised using pre-processed datasets of fingerprint images, and its robustness is validated using three datasets, with 70% for training and 30% for testing. By fine-tuning and optimizing the SAE with L2 and sparsity regularization, the efficiency of learning representation is improved. The results demonstrate that the proposed approach significantly enhances the quality of reproduced fingerprint images by capturing distinct ridge structures and eliminating overlapping patterns [40].

## 3. Methodology

### 3.1. Study Design

**Figure 1:**
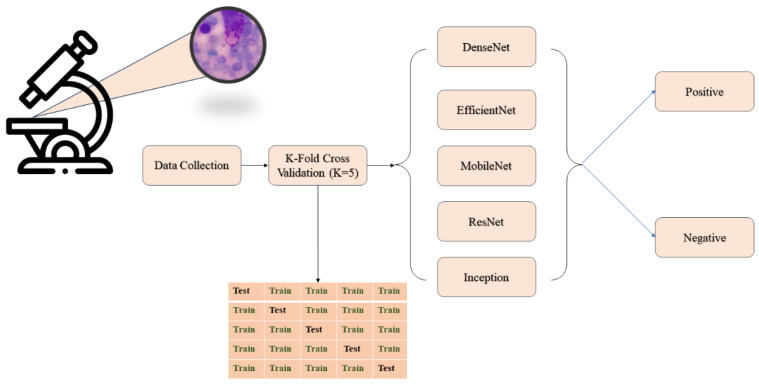
The block diagram of the proposed system.

This study aimed to develop a cutaneous leishmania parasite diagnosis system using the images observed under a microscope. Considered pre-trained models, including MobileNet-v2, Xception, DenseNet-201, ResNet-101, and EfficientNet-b0, as shown in Figure 1.

### 3.2. Data Preparation

A prospective cohort research project was undertaken in the Al-Murqub regions (8841.08 square kilometers) 32°19′12.7″ N 13°57′39.2″ E in the northwestern part of Libya, which encompass five cities and roughly nine villages where ZCL is endemic. The samples have been collected in August, September, and October 2022. Furthermore, the Ethical Committee at Emhammed Almgarif Health Center, situated in the Al-Murqub district, provided approval to conduct the research (EMHC. REF. 22.08.1063).

**Figure 2:**
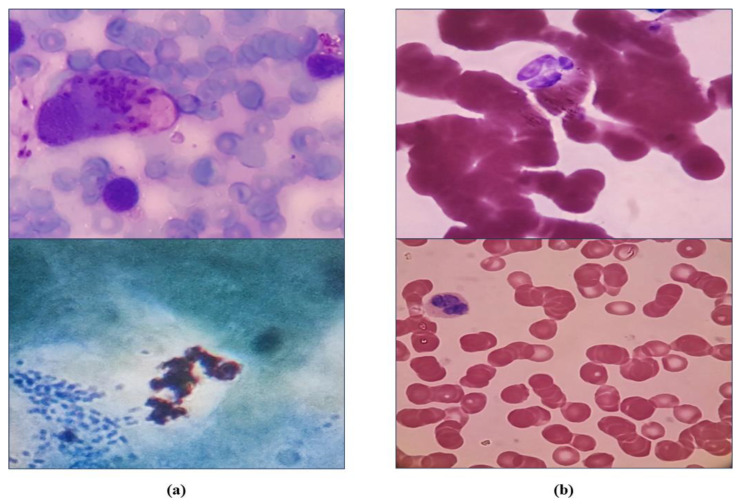
(**a**): cutaneous Leishmania positive. (**b**): cutaneous Leishmania negative.

Infectious disease and clinical microbiology physicians, in conjunction with medical microbiologists, conducted the classification of all visual depictions, including both positive and negative representations. Some of these images are shown in Figure 2.

### 3.3. Pre-Trained Models

The MobileNet-v2, Xception, DenseNet-201, ResNet-101, and EfficientNet-b0 models, which have been trained on a vast dataset and are renowned for their excellent performance in computer vision tasks, were utilised in this study. By utilizing their pre-trained weights, training time is minimised, and model accuracy is enhanced. Furthermore, the models have undergone fine-tuning on the particular dataset employed in this experiment to optimise their performance even more.

#### 3.3.1. DenseNet-201

DenseNet-201 is a 201-layer deep convolutional neural network. It provides a pre-trained variation that was trained on over 1 million images obtained from the ImageNet collection. This previously trained machine learning algorithm is capable of classifying images into 1000 separate object categories, which include items such as mice, keyboards, pencils, and numerous animals. As a result, the network has developed detailed feature representations that cover a wide range of image types. It uses an input picture size of 224 by 224 pixels [41].

#### 3.3.2. Xception

Xception, an acronym for extreme inception, is a convolutional neural network (CNN) model developed by the Google team. Similar to other deep CNNs, this model utilises depth-wise separable convolution and shortcuts between convolutional blocks. However, in the case of Xception, the order of depthwise and pointwise convolutions is reversed compared to MobileNet. In other words, the Xception model applies pointwise convolution before depthwise convolution. The architecture of Xception consists of three components: entry, middle, and exit flows [42].

#### 3.3.3. MobileNet-v2

MobileNet-v2 is designed to be as efficient as possible by employing depthwise separable convolutions, inverted residual blocks, and linear bottlenecks. It is a convolutional neural network with 53 layers of depth. This network can be launched with a pre-trained model that was trained on a huge dataset of over one million photographs from ImageNet. This pre-trained model can classify images into over 1000 different object categories, including keyboards, mice, pencils, and several animal species. Notably, the network operates with picture inputs of 224 by 224 pixels [43].

#### 3.3.4. ResNet-101

ResNet-101 is a well-known model in the field of computer vision that was created to meet issues in image identification jobs. This model has a complex network structure with 104 convolutional layers organised into 33 layers blocks. Twenty-nine of these blocks are physically connected to the ones before them, producing a hierarchy of interconnected levels. Extensive empirical research has shown that these residual networks have a higher degree of optimization ease and may successfully exploit increased depth to produce improved accuracy. These findings have been validated by thorough experimentation on the ImageNet dataset, confirming ResNet-101′s applicability and resilience in the field of computer vision [44].

#### 3.3.5. EfficientNet-b0

The EfficientNet-b0 model is well-known for its smart and all-encompassing network design. It distinguishes itself by obtaining a phenomenal top-1 accuracy of 84.3% on the difficult ImageNet dataset while also delivering considerable efficiency benefits. Despite its efficiency, EfficientNet contains a large number of variables, approximately 66 million and conducts nearly 37 billion floating-point operations per second (FLOPS). EfficientNet is around 8.4 times more compact and has 6.1 times quicker prediction performance when compared to the most sophisticated convolutional neural networks (CNNs) obtainable [45].

### 3.4. Experimental Design

The configuration of the computer used to perform the experiments is 32 GB of RAM, an NVIDIA GeForce RTX 2080-Ti graphics processor, and an i9-9th Generation CPU. Each model is trained using the Adam optimiser while maintaining a batch size of 10 and a learning rate of 0.001. Additionally, all models underwent training for a maximum of 30 epochs.

### 3.5. Performance Evaluation

The performances of the models are evaluated using Receiver Operating Characteristic (ROC) curve analysis. In this analysis, the area under the curve (AUC) was calculated to measure the overall model accuracy in both the training and validation sets. Additionally, we assessed the model performance using metrics such as sensitivity, specificity, precision, Matthew’s correlation coefficient (MCC), and Cohen’s kappa and F1-score to provide a comprehensive evaluation of its effectiveness.

Accuracy is a metric that quantifies the proportion of right predictions to determine how accurately an algorithm predicts outcomes.

(1) $$Accuracy=\frac{TP+TN}{TP+TN+FP+FN}$$

Sensitivity, often known as recall, assesses an algorithm’s ability to recognise genuine positives within all positive cases.

(2) $$Sensitivity=\frac{TP}{TP+FN}$$

Precision calculates the ratio of true positives compared to all predicted positives to determine the accuracy of an algorithm’s positive predictions.

(3) $$Precision=\frac{TP}{TP+FP}$$

Specificity, also known as the true negative percentage, is the number of actual negative observations correctly estimated by an algorithm out of all negative occurrences.

(4) $$Specificity=\frac{TN}{TN+FP}$$

The F1 score is an indicator that combines precision and recall to provide a balanced evaluation of a model’s accuracy for positive as well as negative predictions. The weighted harmonic mean of these two metrics is used to calculate it.

(5) $$F1\;score=2\times \frac{Precision\times recall}{precision+recall}$$

The Matthews correlation coefficient (MCC) evaluates the effectiveness of a binary classification model on a scale of −1 to +1. A −1 score indicates an inadequate classifier, while a +1 score indicates an accurate classifier. MCC is regarded as a well-balanced metric because it considers both positive and negative results.

(6) $$MCC=\frac{\left(TP\times TN\right)-(FP\times FN)}{\sqrt{\left(TP+FP)(TP+FN)(TN+FP)(TN+FN\right)}}$$

Cohen’s kappa is a statistic used to quantify the level of agreement between a model’s overall accuracy and the accuracy expected by chance. The kappa value, which is determined using Equation (7), falls between 0 and 1. A kappa value of 0 implies no agreement, whereas a kappa value of 1 shows complete agreement.

(7) $$K=\frac{P_{o}-P_{e}}{1-P_{e}}$$

where P_o_ is the accuracy of the model (Equation (1)), and P_e_ is the hypothetical probability of chance agreement, computed as:

(8) $$P_{e}=\frac{\left(TP+FN\right)\left(TP+FP\right)+(FP+TN)(FN+TN)}{{\left(TP+TN+FP+FN\right)}^{2}}$$

## 4. Results and Discussion

The healthcare system is transforming to improved accuracy, speed, and reliability due to the integration of AI-based techniques. In diagnosis, supervised and unsupervised machine learning models are being utilised to enable automated, intelligent diagnosis by healthcare professionals. Over the past decade, numerous deep learning models, including CNNs and ANNs, have been developed and implemented in healthcare to assist in diagnosing various diseases.

In recent years, deep learning models have been created and applied for the detection of several diseases. This study explored five pre-trained deep learning models for binary classification of microscopic images into positive and negative cutaneous leishmaniasis.

### 4.1. Results

The results presented are based on the mean performance across five-fold cross-validation. The classification performance measures for cutaneous leishmaniasis using the five different pre-trained deep learning models with five-fold cross-validation are shown in Table 1, Table 2, Table 3, Table 4 and Table 5.

**Figure 3:**
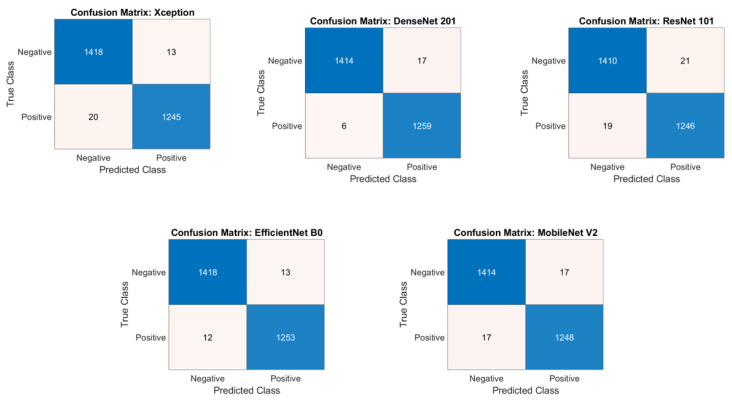
Confusion Matrix for the classification of microscopic images using pre-trained models.

**Figure 4:**
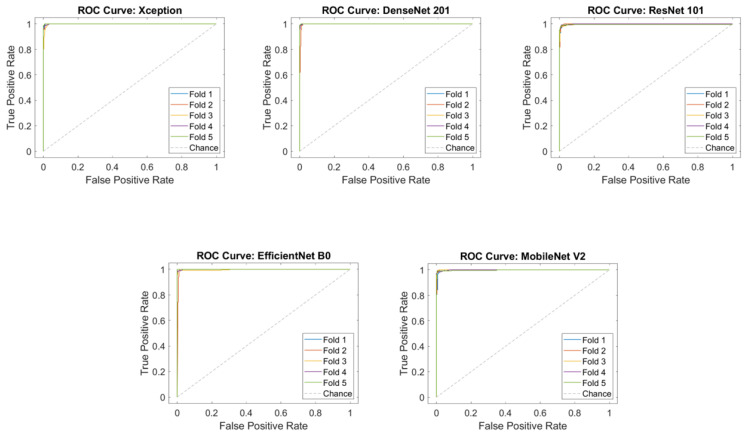
AUC score of pre-trained models.

The AUC scores and confusion matrices for the classification of microscopic images using pre-trained deep-learning models are presented in Figure 3 and Figure 4, respectively.

**Figure 5:**
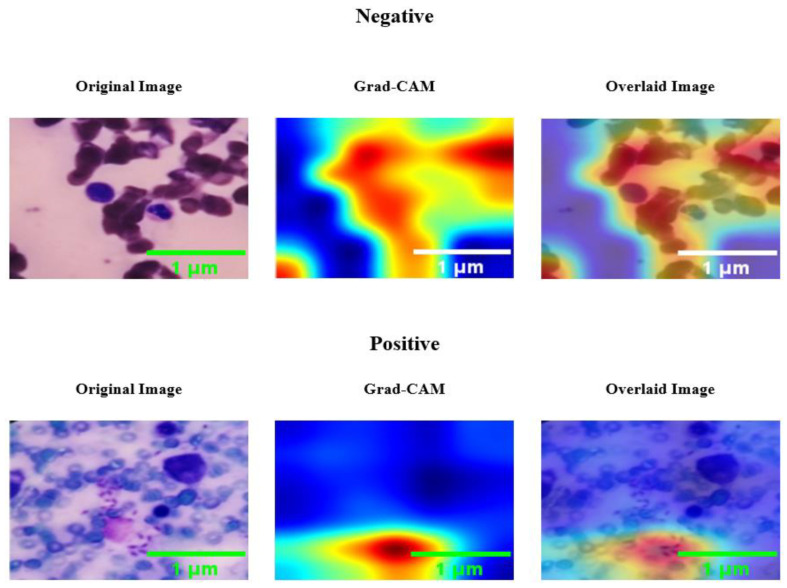
Original image, CAM, and Overlaid image for each class.

Figure 5 demonstrates the Class Activation Map (CAM) examples for both classes generated by the considered models.

Table 1, Table 2, Table 3, Table 4 and Table 5 demonstrate the results of the considered deep learning models when applied to microscopic images of the cutaneous leishmanial amastigote stage. The proportion of properly identified cases is measured by accuracy, whereas the F1-score combines precision and recall to provide a balanced assessment of model performance. The Matthews correlation coefficient (MCC) and the Cohen’s kappa coefficient were employed as assessment measures.

DenseNet-201 model exhibited notable performance by achieving an accuracy of approximately 0.99146911 and an F1 score of about 0.99102. These metrics suggest that the model accurately classified 99.14% of the samples in the dataset while maintaining a commendable balance between precision and recall, as evidenced by the high F1 score. EfficientNet-b0, while slightly trailing behind DenseNet-201 in terms of accuracy with an achievement of approximately 0.990727, demonstrated a strong F1 score of about 0.990129. This signifies that the model maintained an excellent trade-off between precision and recall, highlighting its robust classification capabilities. MobileNet-v2 showcased admirable performance, with an accuracy of about 0.98738748 and an F1 score of approximately 0.986638. Despite slightly lower accuracy than the aforementioned models, MobileNet-v2 still managed to classify 98.74% of the samples accurately and upheld a high F1-score, signifying its effectiveness in classification tasks. ResNet-101 delivered robust results, with an accuracy of around 0.9851632 and an F1 score of about 0.984165. This model’s high accuracy and balanced F1 score underscore its proficiency in accurate sample classification. Xception, akin to MobileNet-v2, achieved commendable accuracy, approximately 0.987757163, and a well-balanced F1-score of about 0.986926. This outcome reflects Xception’s effectiveness in the classification task, as it maintained a high level of accuracy and a harmonious balance between precision and recall.

The results of various deep learning models on microscopic images of the cutaneous leishmanial amastigote stage. The assessment metrics utilised, the Matthews correlation coefficient (MCC) and Cohen’s kappa coefficient, provide information about the agreement between projected and actual classifications. DenseNet-201 outperformed the other topologies, with an MCC of 0.98302 and a Cohen’s kappa coefficient of 0.98289. With an MCC of 0.98148 and a Cohen’s kappa coefficient of 0.98136, EfficientNet-b0 came in second. These results reveal that both designs had high agreement in identifying the images, demonstrating their effectiveness in this task.

### 4.2. Comparison of the Model Performances

The evaluation and comparison of model performance using a five-fold cross-validation approach has revealed significant findings: the DenseNet-201achieved superior results in terms of accuracy, sensitivity, NPV, MCC, F1-Score, and Cohen’s Kappa is 0.9914691, 0.9952845, 0.9958081, 0.99102, 0.98302 and 0.98289, respectively. In terms of specificity and AUC, Xception achieved the best results with 0.990890819 and 0.999481, respectively. EfficientNet-b0 achieved the best result in terms of PPV with 0.9901014. This shows that DenseNet-201 achieved the best result, followed by Xception, as shown in Table 6.

### 4.3. Discussion

When we compared our findings to those of prior research on the amastigote stage of CL, Górriz’s [30] research focused on the promastigote and amastigote stages of *Leishmania infantum*, *Leishmania major*, and *Leishmania braziliensis*, and they used the U-Net model to train. A model had a precision of 0.757, which means that 75.7% of the predicted amastigote of cutaneous leishmania occurrences were right. With a recall of 0.823, the model correctly detected 82.3% of the actual amastigote occurrences. Zare et al. [31] created The Viola–Jones approach algorithm with an adaboost optimiser algorithm using a dataset of 300 images of positive and negative cutaneous leishmaniasis. The results showed that detecting macrophages infected with leishmania parasites had a 65% recall and 50% precision, and identifying amastigotes outside of macrophages had a 52% recall and 71% precision. Limon Jacques [32] captured microscopic images with a smartphone at a magnification of 50 were pre-processed and subjected to pre-training using the K-means algorithm, histogram thresholding, and the U-net structure for segmenting promastigotes and amastigotes forms in cutaneous leishmaniasis. The precision and recall values for amastigotes stages were 61.07% and 87.90%, individually, based on the segmentation data. However, the precision and recall scores for promastigotes were 91.0% and 47.14%, respectively.

In the study conducted by Maqsood et al. [46], experimental evaluations are performed on the benchmark NIH Malaria Dataset, and the results reveal that the proposed Xception model is 0.9494% accurate and 0.9494% F1-score in detecting malaria from the microscopic blood smears. Meanwhile, Densenet-201 achieved 0.9054% accuracy and 0.9052% F1-score. Biswal et al. [47] conducted an experiment with the MobileNet-v2 neural network model on a Kaggle dataset of 12,444 augmented images exhibiting diverse blood cell types classified into four separate classes. Pre-processing processes included data refining and image resizing to a consistent size of 128 × 128 pixels. In the study, two adaptive optimization methods, Adam and stochastic gradient descent (SGD), were used. The Adam optimiser produced an accuracy of 0.920, while the SGD optimiser provided an accuracy of 0.90, reflecting how well the model performed in categorizing the blood cell images. The research was carried out by Hiremath [48]; an investigation was conducted to assess the efficacy of various models for the classification of histopathological breast cancer images into benign and malignant categories at different magnification levels, specifically at 40×, 100×, 200×, and 400×. The models employed for this task included EfficientNet-b0 and EfficientNet with HSV colour transformation. The research utilised an openly accessible dataset sourced from Kaggle, comprising a total of 7909 images, with 2480 representing benign cases and 5429 representing malignant cases. The performance evaluation of EfficientNet-b0 was based on the accuracy metric, resulting in classification accuracy rates of 86%, 88%, 88%, and 83% for the respective magnification levels. Xu and his colleagues [49] analysed a dataset consisting of 4011 IVCM images captured from a total of 48 eyes. These eyes were categorised into different groups, including 35 eyes with keratitis, 7 eyes with dry eyes, and 6 eyes with pterygium. The original IVCM images were standardised to a resolution of 224 × 224 pixels. Deep Transfer Learning was conducted using various neural network models, with one of them being Residual Network-101. The findings revealed that ResNet-101 exhibited a notable level of accuracy, achieving a score of 0.9283.

## 5. Conclusions

To summarise, the top-performing deep learning models for identifying microscopic images of the cutaneous leishmanial amastigote stage were DenseNet-201 and EfficientNet-b0. DenseNet-201 displayed a well-balanced trade-off between precision and recall, demonstrated by its high F1 score and an accuracy of roughly 99.14%. It also had a high level of agreement with actual classifications, as evidenced by its MCC and Cohen’s kappa coefficient. EfficientNet-b0 followed closely behind with an accuracy of approximately 99.07% and comparable performance in terms of F1 score, MCC, and Cohen’s kappa coefficient. All models demonstrated their ability to effectively categorise the data while maintaining a harmonious mix of precision and recall.

Finally, when the deep learning models are tested using microscopic images of the cutaneous leishmanial amastigote stage, DenseNet-201 and EfficientNet-b0 excelled at accurately identifying the samples, displaying good agreement with the real classifications. These models demonstrated strong classification abilities while maintaining a commendable combination of precision and recall. However, when choosing the best model for practical applications, it is critical to examine the specific requirements and limits of the given task.

## Figures and Tables

**Table 1:** Displays the average performance of the DenseNet201 design.

DenseNet-201									
Folds	ACC	SV	SP	PPV	NPV	F1-Score	MCC	CK	AUC
Fold1	0.9888683	1	0.979021	0.976834	1	0.98828	0.977927	0.977683	0.999820
Fold2	0.9907407	1	0.9823322	0.980916	1	0.99037	0.981624	0.981455	0.997786
Fold3	0.9907236	1	0.9825175	0.9806202	1	0.99022	0.981568	0.981399	0.999931
Fold4	0.9907236	0.9847909	0.9963768	0.9961538	0.9856631	0.99044	0.981492	0.981431	0.999766
Fold5	0.9962894	0.9916318	1	1	0.9933775	0.9958	0.992504	0.992476	0.999944
Average	0.9914691	0.9952845	0.9880495	0.9869048	0.9958081	0.99102	0.98302	0.98289	0.999450

**Table 2:** Demonstrates the typical productivity of the EfficientNet-b0 model implementation.

EfficientNet-b0									
Folds	ACC	SV	SP	PPV	NPV	F1-Score	MCC	CK	AUC
Fold1	0.9981447	1	0.9965035	0.996063	1	0.99803	0.99628	0.99628	1.000000
Fold2	0.9907407	1	0.9823322	0.980916	1	0.99037	0.98162	0.98146	0.996322
Fold3	0.9907236	0.9841897	0.9965035	0.996	0.9861592	0.99006	0.98143	0.98136	0.997609
Fold4	0.9851577	0.9923954	0.9782609	0.9775281	0.9926471	0.98491	0.97042	0.97031	0.999656
Fold5	0.9888683	0.9748954	1	1	0.9803922	0.98729	0.97764	0.97739	1.000000
Average	0.990727	0.9902961	0.99072	0.9901014	0.9918397	0.99013	0.98148	0.98136	0.998717

**Table 3:** Exemplifies the standard productivity achieved through the implementation of the MobileNet-v2 model.

MobileNet-v2									
Folds	ACC	SV	SP	PPV	NPV	F1-Score	MCC	CK	AUC
Fold1	0.9814471	0.9841897	0.979021	0.9764706	0.9859155	0.98031	0.9628	0.96277	0.998231
Fold2	0.9907407	0.9844358	0.9964664	0.996063	0.986014	0.99022	0.98149	0.98143	0.999237
Fold3	0.9925788	0.9960474	0.9895105	0.9882353	0.9964789	0.99213	0.98514	0.98511	0.999572
Fold4	0.9814471	0.9847909	0.9782609	0.9773585	0.9854015	0.98106	0.96291	0.96288	0.999187
Fold5	0.9907236	0.9832636	0.9966667	0.9957627	0.9867987	0.98947	0.98124	0.98118	0.998075
Average	0.9873875	0.9865455	0.9879851	0.986778	0.9881217	0.98664	0.97471	0.97467	0.998860

**Table 4:** Shows the standard performance attained by implementing the ResNet-101 model.

ResNet-101									
Folds	ACC	SV	SP	PPV	NPV	F1-Score	MCC	CK	AUC
Fold1	0.9795918	0.9644269	0.993007	0.9918699	0.9692833	0.97796	0.95929	0.95896	0.998784
Fold2	0.9851852	1	0.9717314	0.9698113	1	0.98467	0.97077	0.97034	0.998790
Fold3	0.9814471	0.9802372	0.9825175	0.9802372	0.9825175	0.98024	0.96275	0.96275	0.998452
Fold4	0.9907236	0.9923954	0.9891304	0.9886364	0.9927273	0.99051	0.98144	0.98144	0.999160
Fold5	0.9888683	0.9874477	0.99	0.9874477	0.99	0.98745	0.97745	0.97745	0.995328
Average	0.9851632	0.9849014	0.9852773	0.9836005	0.9869056	0.98417	0.97034	0.97019	0.998103

**Table 5:** Illustrates the mean level of performance achieved by employing the Xception model.

Xception									
Folds	ACC	SV	SP	PPV	NPV	F1-Score	MCC	CK	AUC
Fold1	0.987012987	0.976284585	0.996503497	0.995967742	0.979381443	0.986028	0.974068	0.973898	0.999820
Fold2	0.994444444	0.996108949	0.992932862	0.992248062	0.996453901	0.994175	0.988872	0.988865	0.999698
Fold3	0.975881262	0.960474308	0.98951049	0.987804878	0.965870307	0.973948	0.951828	0.951504	0.998480
Fold4	0.987012987	0.988593156	0.985507246	0.984848485	0.989090909	0.986717	0.97402	0.974013	0.999476
Fold5	0.994434137	1	0.99	0.987603306	1	0.993763	0.988801	0.988738	0.999930
Average	0.987757163	0.9842922	0.990890819	0.989694495	0.986159312	0.986926	0.975518	0.975404	0.999481

**Table 6:** Comparison of pre-trained model performances.

Models	ACC	SV	SP	PPV	NPV	F1-Score	MCC	CK	AUC
DenseNet-201	0.9914691	0.9952845	0.9880495	0.9869048	0.9958081	0.99102	0.98302	0.98289	0.999450
EfficientNet-b0	0.990727	0.9902961	0.99072	0.9901014	0.9918397	0.99013	0.98148	0.98136	0.998717
MobileNet-v2	0.9873875	0.9865455	0.9879851	0.986778	0.9881217	0.98664	0.97471	0.97467	0.998860
ResNet-101	0.9851632	0.9849014	0.9852773	0.9836005	0.9869056	0.98417	0.97034	0.97019	0.998103
Xception	0.987757163	0.9842922	0.990890819	0.989694495	0.986159312	0.986926	0.975518	0.975404	0.999481

## Data Availability

The data available upon request.
